# Defining sustainability in practice: views from implementing real-world innovations in health care

**DOI:** 10.1186/s12913-020-4933-0

**Published:** 2020-02-04

**Authors:** Robin Urquhart, Cynthia Kendell, Evelyn Cornelissen, Laura L. Madden, Byron J. Powell, Glenn Kissmann, Sarah A. Richmond, Cameron Willis, Jackie L. Bender

**Affiliations:** 1 Department of Surgery, Dalhousie University, Room 8-032, Centennial Building, 1276 South Park Street, Halifax, Nova Scotia B3H 2Y9 Canada; 2 Nova Scotia Health Authority, Halifax, Nova Scotia Canada; 3 Department of Community Health & Epidemiology, Dalhousie University, Halifax, Nova Scotia Canada; 4 Department of Family Practice, Faculty of Medicine, University of British Columbia, Vancouver, BC Canada; 5 Brown School, Washington University in St. Louis, St. Louis, Missouri USA; 6 Interior Health, Kelowna, BC Canada; 7 Health Promotion, Chronic Disease and Injury Prevention, Public Health Ontario, Toronto, ON Canada; 8 The Movember Foundation, Melbourne, Australia; 9 Department of Supportive Care, Princess Margaret Cancer Centre, ELLICSR Cancer Rehabilitation and Survivorship, Toronto, Canada; 10 Dalla Lana School of Public Health, University of Toronto, Toronto, Canada

**Keywords:** Sustainability, Innovations, Qualitative research

## Abstract

**Background:**

One of the key conceptual challenges in advancing our understanding of how to more effectively sustain innovations in health care is the lack of clarity and agreement on what sustainability actually means. Several reviews have helped synthesize and clarify how researchers conceptualize and operationalize sustainability. In this study, we sought to identify how individuals who implement and/or sustain evidence-informed innovations in health care define sustainability.

**Methods:**

We conducted in-depth, semi-structured interviews with implementation leaders and relevant staff involved in the implementation of evidence-based innovations relevant to cancer survivorship care (*n* = 27). An inductive approach, using constant comparative analysis, was used for analysis of interview transcripts and field notes.

**Results:**

Participants described sustainability as an ongoing and dynamic process that incorporates three key concepts and four important conditions. The key concepts were: (1) continued capacity to deliver the innovation, (2) continued delivery of the innovation, and (3) continued receipt of benefits. The key conditions related to (2) and (3), and included: (2a) innovations must continue in the absence of the champion or person/team who introduced it and (3a) adaptation is critical to ensuring relevancy and fit, and thus to delivering the intended benefits.

**Conclusions:**

Participants provided a nuanced view of sustainability, with both continued delivery and continued benefits only relevant under certain conditions. The findings reveal the interconnected elements of what sustainability means in practice, providing a unique and important perspective to the academic literature.

## Background

Despite an increasing emphasis on designing and testing strategies to effectively move evidence-based innovations (i.e., new ideas, technologies, and practices [1]) into healthcare practice and policy, research continues to uncover evidence-to-practice gaps across healthcare settings, conditions, and jurisdictions [2–5]. Clearly, the implementation of innovations in health care is a complex and dynamic process. We also know that new knowledge and tools are often put into practice but their use and/or benefits are not sustained [6]. That is, they do not become integrated into the long-term routines of organizations [7–9] and, as a result, patients do not benefit from the best care possible [10–12].

To date, the vast majority of research in this area has focused on the adoption (i.e., the “decision to make full use of an innovation as the best course of action available” [13]) and early implementation of innovations, and not on their sustained use [14, 15]. In fact, sustainability has been described as “one of the least understood and most vexing issues for implementation research” [16]. From a health services/system perspective, this limited understanding is a major knowledge gap since policy-makers, funders, and other stakeholders are interested in understanding and maximizing the long-term impact of their investments. One of the key conceptual challenges in advancing our understanding of how to more effectively sustain innovations is the lack of clarity and agreement on what sustainability actually means [6, 17, 18]. A standard definition is needed by practitioners and researchers to guide sustainability planning, and to inform evaluation efforts through the operationalization of sustainability outcomes and the development and application of psychometrically strong and pragmatic measures.

Whereas others have proposed definitions based on concepts related to sustainability identified within the existing literature [18–20], this study aims to identify how individuals involved in the implementation and/or sustainment of evidence-informed innovations in health care define sustainability. This knowledge can help us create a standard definition of sustainability that acknowledges and incorporates the perspectives of those working directly to implement and/or sustain innovations.

## Methods

This inquiry was part of a larger mixed-methods study to examine sustainability processes, influences, and measures in cancer survivorship care. Specifically, we conducted a concurrent mixed methods study [21] focusing on the sustainability of evidence-based innovations in cancer survivorship care that were implemented across Canada. Cancer survivorship care aims to address the physical, psychosocial, and economic sequelae of a cancer diagnosis and its treatment, and includes issues related to health care delivery, access, and follow-up care [22]. The larger study was informed by our related research [23–31], Scheirer’s work on sustainability [32, 33], the dynamic sustainability framework (DSF) [34], and the program sustainability framework (PSF) [35]. The DSF proposes the “fit” between the innovation and the setting is key to sustainability, and emphasizes the ongoing adaptation of innovations as they are sustained. The PSF presents nine domains deemed critical to developing and sustaining public health programs, including political support, funding stability, partnerships, and program adaptation. Ethical approval was obtained from the Nova Scotia Health Authority Research Ethics Board.

### Participants

Participants were multi-level stakeholders (e.g., managers, administrators, program staff, clinicians, and researchers) involved in the implementation and/or sustainment of evidence-based innovations relevant to cancer survivorship care. Prior to identifying individuals to approach, we first identified innovations in survivorship care that were implemented in Canada (e.g., self-management tools, physical activity programs, and models of follow-up care) and were past the initial funding period. Potentially eligible innovations were identified via: authors’ [RU, JLB] knowledge and networks, both researchers focusing on cancer survivorship care; a Web search of all provincial and territorial cancer agencies (or equivalent); a search of citations and work/research activities of all members of the Canadian Cancer Survivorship Research Consortium; and a PubMed search for published papers of Canadian-based innovations in cancer survivorship care. Each innovation was then assessed in terms of its level of evidence. Specifically, an innovation was considered evidence-based if at least one published peer-reviewed study, using either an experimental or quasi-experimental study design, existed to demonstrate improved outcomes for the target population. This criterion was selected because it is the National Cancer Institute’s criterion for Research-Tested Intervention Programs specifically aimed at cancer control and cancer survivorship (versus for therapies or diagnostic tests/procedures) [36]. Maximum variation sampling [37] was then used to achieve variation across evidence-based innovations in terms of target population, innovation type [33], and geographic setting. For the recruitment of individual participants, purposive sampling [38] was employed to identify the implementation leaders and/or staff member(s) who were most directly involved in the implementation and/or sustainment of each of the innovations. These individuals were contacted by email and invited to participate in an interview. Data collection continued until thematic saturation was reached [39].

### Data collection

We conducted in-depth, semi-structured telephone interviews after obtaining informed consent from participants. Each interview lasted approximately 40–60 min and was conducted by a Master’s trained research associate with experience in qualitative methods [LLM]. The interviewer had no prior relationship with any of the participants. The interview guide was developed specifically for this study using practical guidance from Patton [40] and Rubin and Rubin [41] (see Additional file 1 for full guide). For this analysis, we analyzed data related to the question “what does sustainability mean to you?” which was asked prior to any discussion of sustainability concepts/definitions from the literature. All interviews were audio-recorded and transcribed verbatim.

### Data analysis

An inductive approach, using constant comparative analysis, was used for analysis of interview transcripts and field notes [42]. Transcripts were by coded line-by-line by one team member [RU] and subsequently reviewed by a second team member [LLM] who had conducted the interviews and was familiar with the transcripts. These two team members [RU, LLM] used inductive analysis to identify salient concepts and themes related to participants’ perceptions of the defining elements or characteristics of sustainability. Participants’ discussion of determinants or factors perceived to affect sustainability were excluded. We used qualitative analysis software (NVivo) to organize and manage the data. Regular research team meetings were held to review, discuss, and confirm findings. Discrepancies were discussed until consensus was reached.

## Results

Of the 32 people contacted, 27 participated in this study; 2 did not respond to the initial invitation while 3 responded suggesting a more suitable person to contact. Participants were implementation leaders or staff of 25 unique cancer survivorship innovations based in six Canadian provinces (British Columbia, Alberta, Manitoba, Ontario, Quebec, and Nova Scotia), although some innovations were delivered Canada-wide. Broadly, the innovations pertained to four main categories: physical activity programs; psychological support/counselling; transitions programs; and return to life and lifestyle programs. Participants described sustainability as an ongoing and dynamic process that incorporates three key concepts and four important conditions (i.e., nuances or caveats). These are discussed below and presented in Table 1.

**Table 1:** Key concepts and important conditions pertaining to sustainability

Key concepts	Important conditions
1. Continued capacity *to deliver the innovation*	
2. Continued delivery *of the innovation*	2a. Innovations must continue in the absence of a champion or the person/team who introduced it 2b. Sustainability is only germane to innovations that are still needed
3. Continued benefits *for the patient, provider, or health system*	3a. Adaptation is critical to ensuring relevancy and fit, and thus to delivering benefits 3b. Sustainability is contingent on being able to demonstrate benefits

### Continued capacity

Participants continually described sustainability as a process that must encompass a continued capacity to deliver an innovation over time. In fact, continued capacity was the most prevalent theme throughout the dataset. Participants discussed capacity mainly in terms of human, financial, and physical resources. This view was reflected by one participant, who said, “*sustainability refers to resources and that includes personnel resources and space resources. Don’t underestimate that one, because space is very difficult … the personnel, space, and then financial resources*” [P18]. Another participant put it this way:

*Putting in place systems that aren’t going to regress because we run out of money and we can’t do it anymore. So it’s really sort of a fiscal and resource sustainability. … So essentially that something is not dependent on something that could disappear, and that could be an individual, it could be money, it could be a skill set. So that’s what sustainability means to me*. [P9].

### Continued delivery

Participants recognized that sustainability must entail continuation of the innovation: “*sustainability, to me, means, like, of course continuing the program and opportunities for the development of the program*” [P19]. One condition, however, that many participants noted was that (2a) sustainability means an innovation continues in the absence of the champion or person/team who introduced it. As one participant stated, “*my broad definition has always been that the program would be able to be maintained and flourish outside of me*” [P23]. Similarly, another participant said, “*That the program would continue beyond me, beyond any one person*” [P1]. A second condition participants discussed was that (2b) sustainability is only germane to innovations that are still relevant and needed. One participant emphasized this by saying, “*it has to fit, always address the need, always has to be relevant. Once you get out of the business of being relevant, you’re at high risk. People either won’t pay or follow through*” [P20]. Related, participants discussed their view that unnecessary or irrelevant innovations should be discontinued not sustained.

### Continued benefits

Most participants noted the sustained use of any innovation is immaterial in the absence of experiencing the intended benefits. Therefore, from their perspectives, sustainability is not anchored on the continued delivery of a program or service, but rather must produce patient, provider, or health system benefits. One participant said, “*to me it means that we’ve been able to identify how much improvement we’ve made and continue to hold that … over time. So that’s one aspect of sustainability, is sustaining the improvement levels*” [P9]. One condition to this concept was that (3a) adaptation is critical to ensuring relevancy and fit, and thus to continuing to deliver intended benefits. This concept was described by one participant who said, “*it’s an ongoing, dynamic process. It continues to grow and develop and evolve. And there needs to be feedback loops. Things have to work, that’s why the refinement and feedback loops are necessary.*” [P20] Further, participants posited that only when an innovation continues to be delivered and a need for it still exists would the innovation be in position to deliver the intended benefits.

A second condition described by participants was that (3b) sustainability is contingent on being able to demonstrate these benefits. One participant expressed this by saying:

*The other part of the sustainability for me was that there was benefit shown in what we were doing. So combining qualitative and quantitative outcomes to show that that it is a needed service and that it was worthwhile to everyone involved.* [P23].

## Discussion

This study explored how multi-level stakeholders who implement and/or sustain evidence-informed innovations in health care perceive sustainability. We found they define sustainability in terms of three concepts: continued capacity to deliver the innovation, continued delivery of the innovation, and continued receipt of benefits. These concepts align well with the academic literature on this topic [18, 43–45]. At the same time, participants provided a nuanced view of these concepts, with both continued delivery and continued benefits only relevant under certain conditions. These findings reveal the interconnected elements of what sustainability means *in practice*; thus, they extend our understanding of sustainability by providing a unique and important perspective to the academic literature.

Moore et al. [18] recently synthesized concepts and definitions from four previously published knowledge syntheses (drawing on > 200 studies) on sustainability in health care as defined by researchers to develop a comprehensive definition of sustainability. Their definition emphasizes both the continued delivery of a program, clinical intervention, and/or implementation strategies (and/or continued individual behavior change) and the continuation of benefits. It also recognizes a program or individual behavior change may adapt while continuing to produce benefits. While highlighting these concepts, participants in this study also stressed several conditions or caveats to them. For instance, they perceived sustainability as only occurring when an innovation continues after the champion and/or person who introduced it leaves the setting (indeed, in this study, several innovations that appeared well integrated into local cancer care systems ceased to exist once the champion or lead staff person left the organization). This highlights both the great influence of well-respected and compelling individuals [27, 46–49], as well as the inherent risk of relying on lone (or small groups of) individuals when implementing innovations. Secondly, they emphasized that continued benefits may be dependent on adaptation to ensure relevancy and fit. This highlights the importance of the dynamic nature of the intervention and its implementation strategy to accommodate evolving needs, contextual circumstances, and evidence, and lends direct support to the DSF [34]. Further, participants highlighted that the issue of sustainability is only meaningful when innovations are still needed. Scheirer and Dearing [32] raised a similar issue when they asked people to consider whether sustainability is desirable. As they stated, “sustaining a program within an ongoing organization could become a hollow shell of activities perpetuated for their own sakes, especially if benefits for clients are not achieved” (p. 2065). In this study, participants discussed similar sentiments, perhaps demonstrating their practical experiences working within care settings.

Several authors have recently shown the most common definition of sustainability is the continuation or maintenance of an innovation or its activities [18, 43]. Interestingly, the most prevalent element of sustainability discussed in this study was continued capacity, which was not integrated in Moore et al.’s recent comprehensive definition [18]. While capacity is often viewed as an input to sustainability versus an integral component [32, 35], participants clearly viewed maintenance of capacity as critical to an inclusive definition. Theories of sustainability originating from the management and organizational sciences literature may add complementary insight into prevailing definitions. For example, institutional theories view sustainability as a dynamic process wherein organizational members develop and/or adapt organizational routines to ensure an innovation becomes part of everyday activities (i.e., routinization) [50]. At the same time, a gradual adaption of the organizational context to the innovation is required (i.e., institutionalization) to embed any new practice into an organization. Although participants in this study viewed innovation adaptation as paramount, their emphasis on the sustainment of capacity may in fact reflect the need to adapt the organizational context (i.e., capacity) to ensure the sustained use of any innovation. Similarly, while much of the emerging guidance arising from the implementation science literature emphasizes innovation adaptation, it might be that adaptation of organizational principles and practices is equally critical to longer-term sustainability. Thus, this realization may mean that maintained capacity (e.g., funding, staffing, expertise, space, and so on) for an innovation is viewed as a desirable sustainability outcome in and of itself. Thinking about sustainability in this way means implementers must not only consider adaptation of the innovation to optimize fit but also adaptation of the organizational principles and work environment to sustain the requisite work practices.

Finally, participants defined sustainability using both process and outcome concepts and language. In both conceptual and empirical work, sustainability is most often viewed as an outcome with innovation components/activities, benefits, and/or capacity maintained [18, 43, 44], although recent authors have included the ability to adapt and continually improve as important to a sustainability definition [18, 34]. Defining sustainability as an outcome may very well aid monitoring and evaluation [32], yet this view may fail to capture the iterative and dynamic nature of sustainability (proposed by Chambers [34]) and highlighted by participants in this study), including the need to continuously monitor, learn, adapt, and improve [34] and the socio-technical character of implementation or health care in general [51, 52].

There are limitations of this study. First, the small number of participants may limit generalizability. The aim of qualitative research, however, is not to achieve generalizable results but to gain a rich understanding of people’s views and experiences. In this study, participants came from six Canadian provinces (in Canada, health care is administered by the provinces, therefore the findings essentially reflect six different healthcare systems) and were involved in the implementation of 25 different cancer survivorship innovations, maximizing the credibility and confirmability of findings through triangulation of data sources. Moreover, although this study focused on innovations implemented in the area of cancer survivorship, there is no apparent reason why those working in cancer survivorship care would view sustainability differently than those working in other clinical care settings. Thus, the findings should be applicable beyond this clinical setting wherein innovations are similar to those implemented in this study (i.e., innovations related to models of care, or lifestyle and/or psychosocial programs and services). However, they may not be transferrable to other types of innovations such as diagnostic or therapeutic innovations that have different purposes and evidence bases. Second, participants included a wide range of managers, administrators, program staff, clinicians, and researchers involved in the implementation and/or sustainment of innovations in cancer survivorship care. While it is possible that some may have had an understanding of the concept of sustainability influenced by the literature, the data did not indicate or suggest a familiarity with the field.

## Conclusions

This study identified how individuals who implement and/or sustain evidence-informed innovations consider and define sustainability. This knowledge should be incorporated into existing definitions of sustainability to encompass the perspectives and experiences of individuals working directly to implement and/or sustain innovations, and therefore guide research relevant to practitioners, managers, and other decision-makers in health care.

## Supplementary information

**Additional file 1.** Draft interview guide for semi-structured interviews.

## Data Availability

The datasets generated and/or analysed during the current study are not publicly available but are available from the corresponding author on reasonable request.

## Abbreviations

DSF: Dynamic Sustainability Framework
PSF: Program Sustainability Framework
