# Supplementary material for: Defining sustainability in practice: views from implementing real-world innovations in health care
Source: BMC Health Serv Res. 2020 Feb 4;20:87. doi: 10.1186/s12913-020-4933-0 (PMC7001290; doi:10.1186/s12913-020-4933-0)
Supplement: Supplementary file 1 — Additional file 1. Draft interview guide for semi-structured interviews. [file 12913_2020_4933_MOESM1_ESM.doc]

**Additional file 1**

**Draft interview guide for semi-structured interviews.**

Setting the stage

1. Can you tell me a bit about *[Innovation X]*?

Probes

- 1. What does *[Innovation X]* entail? What are its components?
  2. Was there any training or education necessary with the implementation?
  3. Were there any policies put into place?
  4. Were there any additional management needs or positions created?
  5. What year was it introduced?
  6. Was there a pilot period? If so, how long was the pilot period?
  7. How long has it been in use (since then)? Is it still in use?

1. How did you first hear about *[Innovation X]*?
   1. What were your initial thoughts?
2. Why was *[innovation X]* implemented here? What need were you trying to address?
   1. Who identified the need for this intervention *[frontline staff, hospital manager/administrators, government]*?
   2. Who proposed *{Innovation X]* as a means of addressing this need *[frontline staff, hospital manager/administrators, government]*?
   3. Did others perceive this need as well?

Implementation

Now I’d like to talk to you a bit about the implementation of [*Innovation X*].

1. Can you tell me about how *[Innovation X]* was implemented?

Probes

- 1. What was your role in the implementation process?
  2. Who else was involved and what were their roles?
  3. How were the relationships between people during the implementation process?
     1. Examples: pre-existing, strength, coming from within or from outside, trust, respect

1. How different was *[Innovation X]* from the existing practices?
   1. Did these differences/similarities affect the implementation process?
   2. If so, how?
2. Would you say that the implementation of *[Innovation X]* was initially successful?
   1. If so, how?
   2. If not, why not?

When I talk about sustainability of innovations, what does sustainability mean to you?

Sustainability

1. Would you say *[Innovation X]* has been sustained so far? Why or why not?
   1. Was use impacted once the initially training/support ended?
      1. If so how?
      2. If not, why not?
2. Has *[innovation X]* been adapted or modified at all?
   1. If so, in what way?
   2. Why were these modifications needed?
3. What did your program/organization do to support the ongoing use or integration of *[Innovation X]* into routine care?
   - - - Examples: policy or operational changes to integrate *[innovation X]* into normal worker expectations or routines
   1. How did that help with the sustainability of *[Innovation X]*?
   2. Engage: Did your team/program/organization try to engage people in the implementation process or the use of the new innovation?
   3. Execute: Did the implementation process go according to the original plan?
4. What factors do you believe influence the sustainability of *[Innovation X]*?

Probes:

- 1. In what ways?
  2. Both facilitators and barriers.
  3. Characteristics of the innovation?
  4. Characteristics of the people managing/leading/supporting its ongoing use?
  5. Organizational context (ex: staffing, IT, infrastructure, organizational culture, management support, incentives, organizational mandates)?
  6. Broader context (ex: policies, regulations, legal, political, or economic context, patient needs/preferences/characteristics)?

1. Earlier we talked about why *[Innovation X]* was implemented here and the needs it was designed to meet. What would you say has been successful in meeting the needs we talked about?
2. What other impacts has *[Innovation X]* had on your program/organization and the people who work there/with the [I*nnovation*]?
   1. What about on the patients?
3. Has there been an evaluation conducted related to the sustained use and/or impacts of *[Innovation X]*?
   1. Are the findings from those evaluations fed back into [*Innovation X]* and used to adapt it?
      1. If so, how so?
4. What do you think is required to ensure the continued use of *[innovation X]* moving forward?
   1. Examples: attitudes, incentives, removal of specific barriers, financial, human resources, organizational mandates/policies
